# Supplementary material for: Comparative Transcriptome Analysis Reveals an Efficient Mechanism for α-Linolenic Acid Synthesis in Tree Peony Seeds
Source: Int J Mol Sci. 2018 Dec 24;20(1):65. doi: 10.3390/ijms20010065 (PMC6337502; doi:10.3390/ijms20010065)
Supplement: Supplementary file 1 [file ijms-20-00065-s001.zip › Supplementary Figures S1-S5.docx]

**Supporting Figure S1.** Length distribution of unigenes for two *Paeonia* species.

**Supporting Figure S2.** Top-hit species distribution for sequences from two *Paeonia* species. Identified by BLASTX against the NCBI-nr. database.

**Supporting Figure S3.** Comparative distribution of GO terms between the two *Paeonia* species.


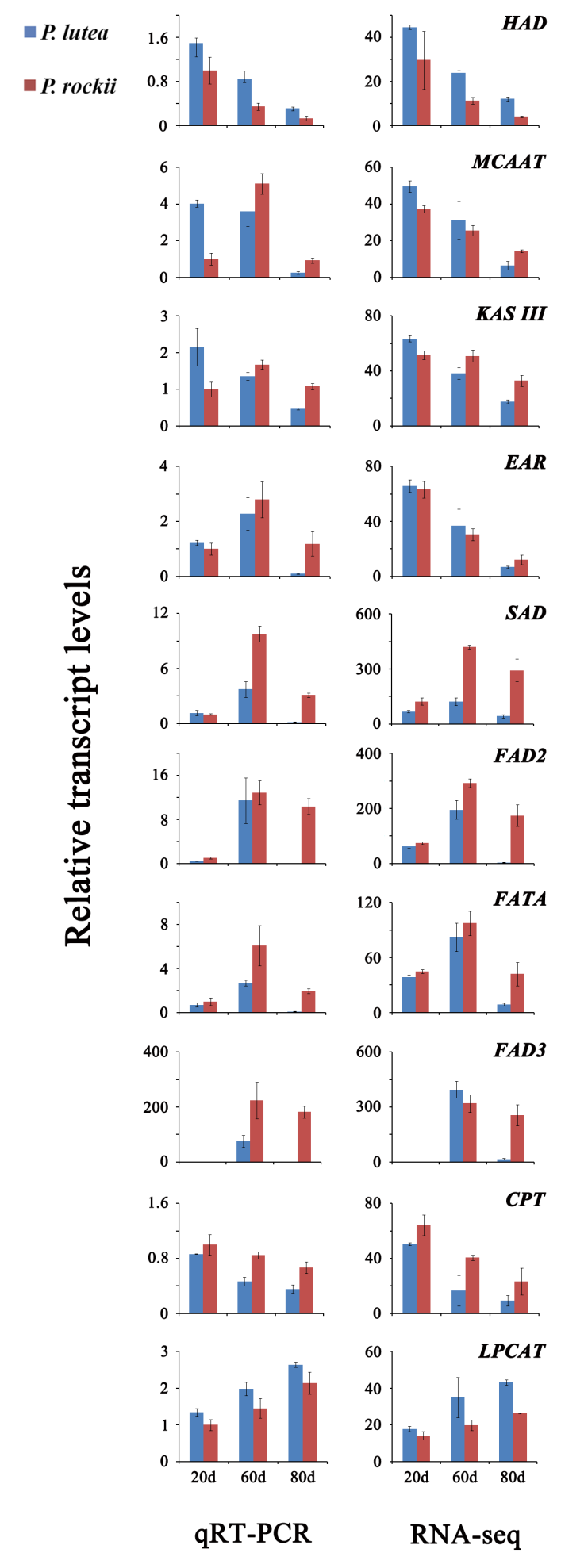


**Supporting Figure S4.** Relative transcript levels of 10 genes by qRT-PCR and RNA-seq between the two *Paeonia* species.


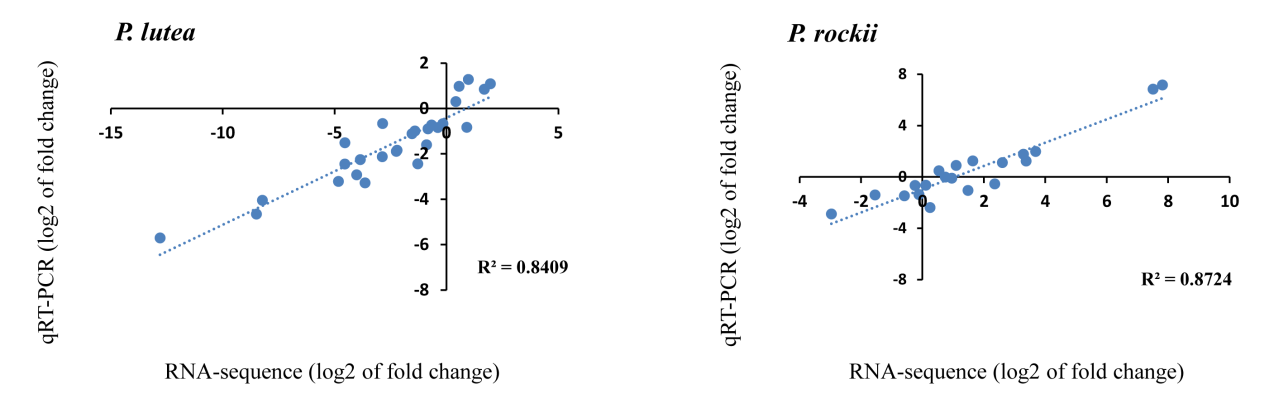


**Supporting** **Figure S5.** Linear regression analysis between qRT-PCR and RNA-seq in *P. lutea* and *P. rockii*.
